# Cohesive Shred Gold, Lamms'

**Published:** 1867-05

**Authors:** 


*Cohesive Shred Gold, Lamms'*.—After several month suse of this preparation of gold, we are disposed to regard it with some favor. Under manipulation, it apparently becomes very dense, and the facility with which it can be used renders it somewhat preferable to crystal or sponge gold. Sufficient time, however, has not elapsed since we commenced the use of it, to enable us to form a correct opinion in regard to its durability, &c.

It certainly does possess great cohesiveness, and when the same care is taken in introducing and condensing it, that is necessary in the use of adhesive foil, there is good reason to believe that a reliable filling may be made of this material.

Its inventor claims for it unequalled softness, cohesiveness and density, and asserts that it condenses and unites *perfectly under fluids*. While we are very willing to bear testimony as to its softness, cohesiveness and density, when introduced in a dry condition, and, from experiments made, of its uniting, in a measure at least, under fluids, yet we do not believe that a *perfect filling* of this, or any other material, can be introduced in a wet condition, not even of "Amalgam." In the centre of several of the blocks of this preparation we have found hard masses, totally unfit for use, and while we are aware of the difficulty of producing in every portion manufactured of this, or of crystal or sponge gold, a perfectly uniform material, yet we think this defect might be remedied if a careful examination was made, and perfect material only be exposed for sale. In using this form of gold, small pieces, from one-half to two-thirds the size of the cavity to be filled, are torn from the block with a fine, sharp pointed instrument, and carried to the position desired, either on the point of the instrument, or by means of small introducing plyers, taking care that the fibres of the gold are not compressed in the handling of it.

The first piece introduced should be firmly fixed, well condensed, and upon this, which serves as a base, the remainder of the filling is built. The manner of introducing and condensing the cohesive shred gold is similar to that of the crystal or sponge gold, care being taken to condense it well against the walls of the cavity. The instruments, however, differ from those used for crystal or sponge gold, their points are not so small, nor so deeply serrated. It is recommended to use tolerably blunt instruments with shallow serrations, somewhat similar, as regards the condensing points, to the ordinary condensers for non-adhesive gold foil.

